# Agreement among Mediterranean Diet Pattern Adherence Indexes: MCC-Spain Study

**DOI:** 10.3390/nu11030488

**Published:** 2019-02-26

**Authors:** Rocío Olmedo-Requena, Carmen González-Donquiles, Verónica Dávila-Batista, Dora Romaguera, Adela Castelló, Antonio José Molina de la Torre, Pilar Amiano, Trinidad Dierssen-Sotos, Marcela Guevara, Guillermo Fernández-Tardón, Macarena Lozano-Lorca, Juan Alguacil, Rosana Peiró, José María Huerta, Esther Gracia-Lavedan, Nuria Aragonés, Tania Fernández-Villa, Marta Solans, Inés Gómez-Acebo, Gemma Castaño-Vinyals, Manolis Kogevinas, Marina Pollán, Vicente Martín

**Affiliations:** 1 Department of Preventive Medicine and Public Health, University of Granada, 18071 Granada, Spain; rocioolmedo@ugr.es (R.O.-R.); macarenalozano@ugr.es (M.L.-L.); 2 Consortium for Biomedical Research in Epidemiology and Public Health (CIBERESP), 28029 Madrid, Spain; vdavb@unileon.es (V.D.-B.); acastello@externos.isciii.es (A.C.); epicss-san@euskadi.eus (P.A.); trinidad.dierssen@unican.es (T.D.-S.); mp.guevara.eslava@navarra.es (M.G.); fernandeztguillermo@uniovi.es (G.F.-T.); juan.alguacil@dbasp.uhu.es (J.A.); rosana.peiro@uv.es (R.P.); jmhuerta.carm@gmail.com (J.M.H.); esther.gracia@isglobal.org (E.G.-L.); nuria.aragones@salud.madrid.org (N.A.); martasolans@gmail.com (M.S.); ines.gomez@unican.es (I.G.-A.); gemma.castano@isglobal.org (G.C.-V.); manolis.kogevinas@isglobal.org (M.K.); mpollan@isciii.es (M.P.); vmars@unileon.es (V.M.); 3 Inst Invest Biosanitaria Ibs GRANADA, 18071 Granada, Spain; 4 The Research Group in Gene-Environment and Health Interactions (GIIGAS), University of León, 24071 León, Spain; ajmolt@unileon.es (A.J.M.d.l.T.); tferv@unileon.es (T.F.-V.); 5 Instituto de Biomedicina (IBIOMED), University of León, 24071 León, Spain; 6 ISGlobal, Centre for Research in Environmental Epidemiology (CREAL), 08003 Barcelona, Spain; dora.romaguera@isglobal.org; 7 Instituto de Investigación Sanitaria de Palma (IdISPa), Hospital Universitario Son Espases, Unidad de Investigación, I-1. Carretera de Valldemossa, 79, 07120 Palma de Mallorca, Spain; 8 CIBER Fisiopatología de la Obesidad y la Nutrición (CIBER-OBN), 28029 Madrid, Spain; 9 Cancer Epidemiology Unit, National Centre for Epidemiology, Instituto de Salud Carlos III, Av/Monforte de Lemos, 5, 28029 Madrid, Spain; 10 Faculty of Medicine, University of Alcalá, Alcalá de Henares, 28801 Madrid, Spain; 11 Public Health Division of Guipuzkoa, BioDonostia Research Institute, 20014 San Sebastian, Spain; 12 Universidad de Cantabria—IDIVAL, 39011 Santander, Spain; 13 Instituto de Salud Pública de Navarra, IdiSNA, 31003 Pamplona, Spain; 14 Instituto de Oncología IUOPA (Instituto Universitario de Oncología del Principado de Asturias), University of Oviedo, 33003 Oviedo, Spain; 15 Centro de Investigación en Recursos Naturales, Salud y Medio Ambiente (RENSMA), Universidad de Huelva, 21071 Huelva, Spain; 16 Fundación para el Fomento de la Investigación Sanitaria y Biomédica de la Comunitat Valenciana FISABIO-Salud Pública, 46020 Valencia, Spain; 17 Department of Epidemiology, Murcia Regional Health Council, IMIB-Arrixaca, 30007 Murcia, Spain; 18 Universitat Pompeu Fabra (UPF), 08003 Barcelona, Spain; 19 Epidemiology Section, Public Health Division, Department of Health of Madrid, 28035 Madrid, Spain; 20 Research Group on Statistics, Econometrics and Health (GRECS), Universitat de Girona, Campus Montilivi, 17003 Girona, Spain; 21 Epidemiology Unit and Girona Cancer Registry, Oncology Coordination Plan, Department of Health, Autonomous Government of Catalonia, Catalan Institute of Oncology, 17004 Girona, Spain; 22 IMIM (Hospital del Mar Medical Research Institute), 08003 Barcelona, Spain

**Keywords:** adherence, Mediterranean diet pattern, indexes, agreement

## Abstract

There are many different methods used to measure the degree of adherence to a Mediterranean diet (MD), limiting comparison and interpretation of their results. The concordance between different methodologies has been questioned and their evaluation recommended. The aim of this study was to evaluate the agreement among five indexes that measure adherence to a Mediterranean dietary pattern. The study population included healthy adults selected in the Multi-Case Control Spain (MCC-Spain) study recruited in 12 provinces. A total of 3640 controls were matched to cases by age and sex. To reach the aim, the following scores of adherence to a Mediterranean dietary pattern were calculated: Mediterranean diet score (MDS), alternative Mediterranean diet (aMED), relative Mediterranean diet (rMED), dietary score (DS) and literature-based adherence score (LBAS). The relative frequency of subjects with a high level of adherence to a MD varied from 22% (aMED index) to 37.2% (DS index). Similarly, a high variability was observed for the prevalence of a low level of MD: from 24% (rMED) to 38.4% (aMED). The correlation among MDS, aMED and rMED indexes was moderate, except for MDS and aMED with a high coefficient of correlation 0.75 (95% CI 0.74–0.77). The Cohen’s Kappa coefficient among indexes showed a moderate–fair concordance, except for MDS and aMED with a 0.56 (95% CI 0.55–0.59) and 0.67 (95% CI 0.66–0.68) using linear and quadratic weighting, respectively. The existing MD adherence indexes measured the same, although they were based on different constructing algorithms and varied in the food groups included, leading to a different classification of subjects. Therefore, concordance between these indexes was moderate or low.

## 1. Introduction

Traditionally, nutritional epidemiology is the study of the relationships between a nutrient or a food and its association with health. Consequently, it is difficult to analyse the effect of a specific food item without considering the rest of the food consumed [1,2,3]. Hence, the study of the dietary patterns has been gaining relevance in the last two decades as it involves evaluating multiple dietary components as a single exposure [4,5,6]. In addition, it is essential to emphasise that diet is a modifiable lifestyle behaviour [7,8].

In 1958, Ancel Keys [9] launched the Seven Countries Study after exploratory research on the relationship between dietary pattern and the prevalence of coronary heart disease in Greece, Italy, Spain, South Africa, Japan, and Finland. He was the first researcher who observed the association between the traditional Mediterranean diet and a low risk of coronary heart disease. The Mediterranean Sea borders 18 countries that differ markedly in geography, economic status, health, lifestyle and diet. However, it is possible to talk about a Mediterranean diet pattern with common characteristics among these countries. Keys defined the Mediterranean diet concept as a model that, instead of highlighting differences among areas, focused on some common features such as a high intake of fruit, vegetables, legumes, nuts and whole grains; olive oil as the main source of added fat; a frequent but moderate intake of red wine at meals; moderate intake of fresh fish, dairy products (specially cheese and yogurt), poultry, meat and eggs; and a low intake in both frequency and quantity of red meat and processed meat [9,10].

At the beginning of the 21st century (i.e., 2003), Trichopoulou et al. [11] published a study describing an overall lower mortality risk in those with the greatest adherence to a Mediterranean dietary pattern. Since then the number of studies carried out on the Mediterranean dietary pattern and its effects on health has increased greatly [12,13,14]. Today we recognise that it is probably due to the combination of many nutrients (for example, vegetables and fruits) with anti-inflammatory, antioxidant and other properties that make the Mediterranean diet recommended for the avoidance of cardiovascular diseases and other illnesses, and therefore, the Mediterranean diet pattern is even referred to as healthy in the 2015–2020 Dietary Guidelines for Americans [15,16]. 

However, measuring the level of adherence to a Mediterranean dietary pattern of a population is not easy. There are different methods used to measure the degree of adherence to a Mediterranean diet and it is precisely this range of methods that is stated as a limitation of the studies that try to test the effects of this dietary pattern on health. Therefore, it is necessary to assess adherence to these dietary pattern indexes and their concordance with the original dietary pattern from the 1950s–60s [17,18]. Most studies that analyse the effect of a Mediterranean dietary pattern on health are predetermined; they use an index constructed from a number of predetermined components according to current nutrition knowledge and specific dietary guidelines [3]. These indexes may also be dependent or independent of the study population, meaning they may be established using the observed distribution of the study samples or may use independent criteria based on general dietary recommendations [19]. Moreover, the considered nutrients and foods included in each index and the score assignment to each subject may also generate variability in the results. Also, as reported in reviews by Bach and Román-Viña et al. [3,4], there are many indexes that measure adherence to the Mediterranean diet pattern, but to date, only one study has assessed the concordance of some of these indexes in a specific population and had a smaller sample size than this study. They concluded that in order to improve the reliability and concordance between the indexes, further studies are required to select the components, the number of components, and the scoring criteria of the indexes to improve their internal consistency [17]. 

For these reasons, the concordance between the different methodologies has been questioned and their evaluation is recommended in order to establish a standard measuring tool [3,17,20]. The aim of this study is to evaluate the agreement among five indexes that evaluate adherence to the Mediterranean dietary pattern.

## 2. Materials and Methods

### 2.1. Study Design, Setting and Participants

The study population were selected among the controls recruited in a multi-case control study in Spain (MCC-Spain). The MCC-Spain is a population-based multi-case control study conducted between September 2008 and December 2013 in 12 Spanish provinces: Asturias, Barcelona, Cantabria, Girona, Granada, Guipúzcoa, Huelva, León, Madrid, Murcia, Navarra, and Valencia (www.mccspain.org). The main aim of the MCC-Spain study was to evaluate the role of environmental exposures and genetic factors on some of the most relevant tumours in the Spanish population: breast, colorectal, gastric, prostate cancer, and chronic lymphocytic leukaemia. Cases were subjects from 20 to 85 years who had been diagnosed and histologically confirmed of any of the previous tumours and accepted to participate. Controls were matched by frequency to cases by age, gender, and region, and were randomly selected from people enrolled in primary care centres within the reference areas of the hospitals where the cases were recruited, and were invited to participate in the study. The protocol of the MCC-Spain study was approved by the ethics committees of the participating centres. All participants were informed about the study objectives and signed an informed consent form. Confidentiality of data was secured by removing personal identifiers in the datasets. The database was registered in the Spanish Agency for Data Protection, number 2102672171. Permission to use the study database will be granted to researchers outside the study group, after revision and approval of each request by the Steering Committee. More details on the organisation of the project can be found online at http://www.mccspain.org. More detailed information can be found in a previous article published by the MCC-Spain project research group [21].

The selection criteria for the MCC-Spain control group were: i) age between 20 to 85 years; ii) resident in the catchment area of the recruiting hospital of the cases during the 6 months prior to the interview; iii) not having a diagnosis of the tumours under study; and iv) be trained to participate in the study. The population of the present study included a subsample of 3640 healthy subjects with dietary information of the total of 4098 subjects selected as controls.

### 2.2. Data Collection

Trained interviewers administered a structured computerised epidemiological questionnaire in a face-to-face interview. Information was collected on socio-demographic and anthropometric characteristics; personal and family background; occupational and residential history; and lifestyle factors including, among others, smoking, alcohol consumption, sleep, and physical activity. Dietary data were collected through a self-administered, semi-quantitative, validated Food Frequency Questionnaire (FFQ) [22].

During the interview, all participants in the MCC-Study received an FFQ in paper form to be completed at home (self-administered) and returned to the interviewer in person or via mail (global response rate 88.8%). The interviewer explained and trained the participant to complete the FFQ. This 140-item questionnaire was a version of an instrument previously validated in Spain [22] modified to include regional products. Collected information referred to eating habits during the preceding year and measured in servings and frequency of consumption each of the foods contemplated in the FFQ. Moreover, some questions about general dietary habits were included in the questionnaire and were used to adjust the responses to the FFQ following the methodology described in Calvert et al. [23]. To facilitate the understanding of some items, photographs were used to assess the level of meat doneness. 

### 2.3. Mediterranean Diet Pattern Adherence Indexes

To meet the objective of this study, the following scores of adherence to a Mediterranean dietary pattern were evaluated:

1. Mediterranean Diet Score (MDS) [11]. This index considers 9 food groups: vegetables, legumes, fruit, fish, cereals, meat, dairy products, monounsaturated/saturated fats ratio and alcohol consumption. The total score ranges from 0 (minimum adherence to a traditional Mediterranean dietary pattern) to 9 (maximum adherence);
2. Alternative Mediterranean Diet (aMED) [24]. In this index the following groups of food/nutrients were considered: vegetables, legumes, fruit, nuts, fish, whole grains, red meat, monounsaturated/saturated fats ratio and alcohol consumption. The total score ranged from 0 (minimum adherence) to 9 (maximum adherence);
3. Relative Mediterranean Diet (rMED) [25]. The following groups of food were considered: vegetables, legumes, fruit, cereals, fish, olive oil, meat, dairy products and alcohol. The total score ranged between 0 (minimum adherence to a traditional Mediterranean dietary pattern) to 18 (maximum adherence);
4. Dietary Score (DS) [26]. This index includes the following groups of food: vegetables, legumes, fruits, fish, whole grains, potatoes, olive oil, poultry, dairy products with fat, red meat and alcohol. The total score ranged from 0 (minimum adherence) to 55 (maximum adherence);
5. Literature-Based Adherence Score (LBAS) [20]: vegetables, legumes, fruits, fish, whole grains, olive oil, dairy products, red meat and processed meat and alcohol. The total score ranged from 0 (minimum adherence) to 18 (maximum adherence).

Table 1 shows detailed information on the characteristics of each of the Mediterranean dietary indexes used in the present study: the basis for its calculation, the groups of food or nutrients included, and the criteria for estimating the final score. The MDS, aMED and rMED scores are indexes that use criteria dependent on the study sample, in contrast to the DS and LBAS indexes whose criteria, rations, grams or energy density are defined a priori. Regardless of the index used, a higher score indicated a higher adherence to a Mediterranean diet pattern. 

### 2.4. Statistical Analysis

Mean, standard deviation (SD) and the 25th, 50th and 75th percentiles were calculated for quantitative variables, and the distribution of absolute and relative frequencies were determined for categorical variables. The participants were classified as having either low, medium or high adherence to a Mediterranean diet according to the cut-off points originally established for these indexes: MDS, aMED and rMED; and LBAS by creating tertiles for the creation of adhesion levels of the DS (see Table 1). Pearson correlation was used to assess the relationship among the scores of the different indexes when they were considered as quantitative variables. A correlation coefficient *R* higher than 0.70 was considered as a strong correlation; from 0.5 to 0.7 as a moderate correlation; and <0.5 as a weak correlation [27,28,29]. When the study population was divided into low, medium and high adherence to a Mediterranean diet according to the criteria used for each index, Cohen’s Kappa coefficient was estimated to measure the concordance between the different indexes using linear and quadratic weighting to penalise classification errors among extreme categories [30]. Cohen’s Kappa coefficient was interpreted according to Landis and Koch classification: fair concordance when the coefficient was ≤0.40; moderate concordance for coefficients from 0.40 to 0.60; and substantial almost perfect concordance when the coefficient was >0.60 [31]. The statistical programme Stata v.14 (Stata Corp., LP, College Station, TX, USA) was used for the data analysis. 

## 3. Results

Of the 4098 healthy controls of the MCC-Spain project, FFQ data were available for 3640 subjects (88.8%). There were no important differences between the population with and without FFQ, except for academic levels (higher response rate for participants with high level studies than for those with primary school education or less, 90.4 versus 84.7%, respectively). The mean age of the participants was 62.8 years (SD 12.0) with an age range between 24 and 85 years. Men made up 50.9% of the whole sample and 21.3% had university level education. The average energy intake of the participants was 1898.3 Kcal/day (SD 638.8). Supplementary Materials Table S1 shows the main socio-demographic characteristics of the subjects included and not included in the analyses.

**Figure 1:**
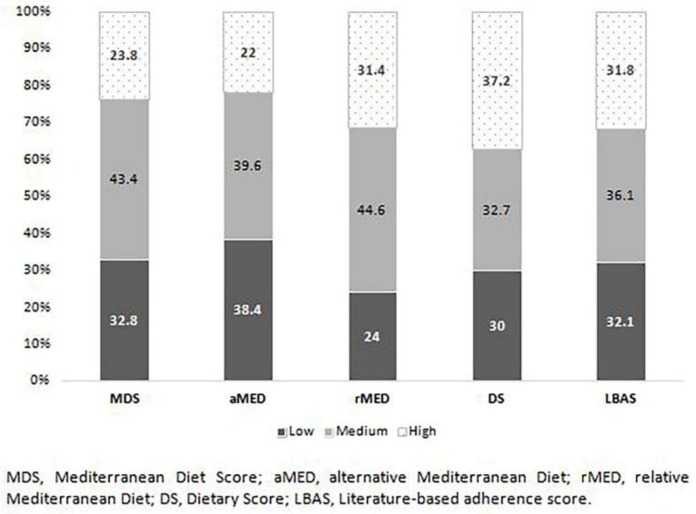
Descriptive analysis of levels of adherence to the Mediterranean diet; rMED, relative Mediterranean diet; DS, dietary score; LBAS, literature-based adherence score.

The mean scores of each of the indexes according their distribution by socio-demographic data is shown in Table 2. Figure 1 shows the distribution of the study population according to the level of adherence to a Mediterranean diet pattern for each one of the indexes. The relative frequency of subjects with a high level of adherence to a Mediterranean diet varied from 22% (95% CI 20.67–23.40) when using the aMED index to 37.2% (95% CI 35.67–38.83) with the DS index. Similarly, a high variability was observed for the prevalence of population with a low level of adherence to a Mediterranean diet, from 24% (95% CI 22.64–25.44) with the rMED index to 38.4% (95% CI 36.81–40.00) with the aMED index. Interestingly, it was only for the DS and LBAS indexes, scores independent of the study population, for which a similar distribution was observed—approximately one-third for each level of adherence. Confidence intervals for proportions to compare the categorical indexes are included in Supplementary Materials Table S2.

Table 3 shows the degree of agreement among the different indexes. A clear pattern of agreement among indexes cannot be observed. The closest agreements are found for the lower adherence categories; and for these categories, aMED is the index with highest agreement with the rest of indexes: from 64.5 with DS to 78.2 with MDS. For categories with higher adherence to a Mediterranean diet, MDS is the index with closer agreements; although they were always lower than 0.70. For the intermediate category, the agreement did not reach 50% for any index.

**Figure 2:**
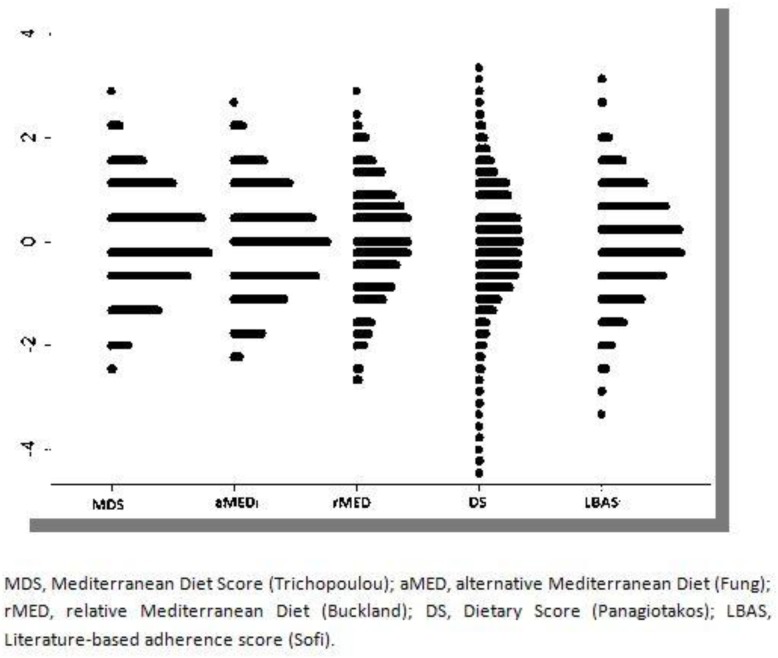
Dotplot representing standardised values of score according to each Mediterranean diet index (*n* = 3640).

Figure 2 shows that, independent of the index used, a normal distribution was observed. Because the possible score range of the DS was higher (0–55 points) compared to the rest of the indices used, the distribution of the subjects in this index was less dense, and consequently, the variability of the score was higher. The agreement between MDS, aMED and rMED indexes was moderate, except for MDS and aMED for which a high correlation was observed, coefficient of correlation 0.75 (95% CI 0.74–0.77). The concordance for DS and MDS, and with the rest of the indexes, was moderate–weak (see Table 4). When the categories of adherence to a Mediterranean diet were considered, the Kappa coefficients among indexes showed a moderate–fair concordance; except for MDS and aMED with a Kappa coefficient of 0.56 (95%CI 0.55–0.59) and 0.67 (95% CI 0.66–0.68) using linear and quadratic weighting, respectively.

## 4. Discussion

Our results show a moderate or low correlation and concordance in the analysed indexes according to the evaluated studies of adherence to Mediterranean diet patterns. As such, a misclassification bias could occur according to the index used. However, we do not have the criteria to decide which index may be better. Therefore, this moderate-to-low correlation should always be considered among the reasons explaining the inconsistent results and variability between studies related to a specific disease and the Mediterranean diet since they can be motivated and explained in part by the use of different indexes to measure adherence to the Mediterranean diet pattern.

### 4.1. Methodological Differences Between Indices

The steep rise in the investigation of the health consequences of a Mediterranean diet pattern has resulted in the proliferation of indexes [32]. These indexes should be comparable with each other. However, disagreement is usual. There are several reasons that may explain the disagreements found: the lack of common criteria to develop the indexes, the type of foods or nutrients considered, the variability of the methods used to construct them, and the dependence or independence of the scores from the study sample. These points are further explained below.

(A) Type of food, base components and/or nutrients included in the indexes. For example, classification agreement for the level of adherence to a Mediterranean diet among indexes is difficult when there are differences in the types of fat included. Differences include: i) monounsaturated/saturated fat ratio are included in MDS and aMED and excluded in the rest of the indexes; ii) dairy products are included in the MDS, rMED, and LBAS indexes but not in the aMED; iii) the type of meat included: all kinds of meat; or only poultry, processed or red meat; iv) cereals are only included in MDS, aMED, and rMED; however, the MDS and rMED included all type of cereals and the aMED includes only whole grain; v) nuts may be considered part of the fruit group as in the MDS or rMED, or like an independent group in the aMED, or not considered at all such as in the LBAS and DS.
(B) Criteria used to build the index. There are indexes based on in g/day (i.e., MDS and LBAS), in rations/day (i.e., aMED) or rations/month (i.e., DS) or in energy density (i.e., rMED). Regardless of how intake is measured, some indexes use scores with established fixed points (i.e., DS and LBAS) and others use sex-specific medians (i.e., MDS and aMED) or tertiles derived directly from the population (see next point). All these differences can be factors that make it difficult to observe a good correlation between the indexes.
(C) Dependence or not on the study sample. While the MDS, aMED and rMED are estimated using the distribution of some food and nutrients in the study sample, the DS and LBAS are based on previously published a priori recommendations. This may explain part of the variability found among indexes. When MDS, aMED or rMED are used, it may occur that if the level of general adherence to the diet of a population is low, when having to classify the population in three levels, there are subjects classified with a medium or high adherence level to a Mediterranean diet when they do not really have it. For example, one participant was classified with a medium adherence with MDS and aMED, but was classified with low adherence for the indexes that did not depend on the population (i.e., DS and LBAS). That is to say, the obtained scores using these indexes for an individual subject are relative and dependent on the characteristics of the rest of the sample, which makes their comparability difficult.

For all the above reasons, the indexes that have more elements in common are those with the best correlation and concordance. In our case, MDS and aMED showed good correlation and substantial–moderate concordance depending on the Kappa used. In fact, differences between these two indexes are minimal and based only on food groups and what foods or nutrients are included in each group. On the other hand, despite having the MDS index as a reference, the rMED has a moderate correlation and a moderate–low concordance with MDS and aMED. 

Bamia et al. [32] evaluated the correlation between the MDS_FFQ index (reference index) and other indexes (Baseline Nutrition Credits4Health (MDS_BNC4H); Mediterranean Diet Index (MDI_BNC4H) and Mediterranean Diet Assessment Score (MEDAS_BNC4H)), and also observed a low-to-moderate magnitude among indexes [32]. Those results are consistent with ours with the correlations and concordances found between the different patterns evaluated in our study also being moderate or low [17,32].

Despite sharing an a priori methodology, DS and LBAS indexes have weak correlation and fair concordance. In this sense, DS is the index which presents the worst correlation and concordance results with the rest of the indexes and this could be explained, in part, due to a larger score range (0–55). Moreover, DS’s method is the one that presents more differences in the number and type of foods and the characteristics those foods must have to be included in the food groups. 

The LBAS index appeared as a proposal to try to standardise all used indexes. This index does not take into account the sample distribution for either the assignment of a score for each food group or level categorisation of adherence to the Mediterranean diet. For our population, LBAS presents better correlation and concordance with MDS and aMED than with rMED, although this good relationship will depend on the population investigated and its particular characteristics. 

As we have observed, the components included in a Mediterranean diet pattern adherence index can vary; consequently, the reliability of the indexes can be lowered. Therefore, the contribution of each component in the indexes, the number of components, and the scoring criteria should be established in order to improve the agreement among indexes. We would consider it essential that all authors use indexes of adherence to the Mediterranean diet with the same characteristics. Thus, by improving these indexes the higher correlations could will lead to stronger evidence of the inverse relation between the Mediterranean dietary pattern and the prevalence of several diseases.

### 4.2. Strengths and Limitations

As strengths of this paper, our results were consistent with those shown in the study by Milà-Villarroel et al. [17] in which they observed low correlations between the indexes. However, their study assessed concordance and reliability in a smaller and selected population of 324 healthy undergraduates at the University of Barcelona, compared to the larger sample and geographical variability of our study. We worked with the control group of MCC-Spain study: 3640 healthy subjects from 12 areas of Spain between the ages of 20 to 85 years. 

Nonetheless, we must mention the limitation of sources and strategies of the collection of dietary information, as it was done using a self-administrated FFQ previously validated for the Spanish population and referred to the intake during the year prior to the interviews [22]. Despite the FFQ not being a perfect method to estimate dietary intake without error, an alternative which lacks limitations does not exist; therefore, the FFQ is considered the reference dietary measurement instrument in nutritional epidemiology, and specifically, in nutrition studies, they allow adequate and replicable estimations of subjects’ dietary habits [33]. However, even though the a priori measurement instrument may be valid, the existence of information biases, particularly recall bias, cannot be ruled out, as well as the fact that participants tend to refer to the frequency with which they consider that they should consume the different types of food and not their current frequency, i.e., bias of social desirability [34]. Furthermore, it is necessary to emphasize that the Spanish Mediterranean diet could be different from that of the rest of the Mediterranean countries, due to the consumption of some local foods which are typical of each region. However, Spain is included in the Mediterranean zone and this food pattern is known as a Mediterranean dietary pattern.

## 5. Conclusions

The results of our study show that the existing Mediterranean diet adherence indexes measure the same concept, although they are based on different constructing algorithms and definitions in the food groups included, leading to a different classification of subjects in the different indexes. This lack of concordance may affect the analysis of the role the Mediterranean diet plays in the health of the population. Concordance between these indexes is moderate or low; therefore, it would be appropriate to reach a consensus on the construction of the indexes to improve the reliability of the results obtained by different indexes. For future research, our proposal would consist of defining which food groups or nutrients are included, and for each group, which particular foods are included, and defining a priori cut-offs because they are scores that do not depend on the general quality of the diet. Lastly, these definitions need to be according to the characteristics of the foods and nutrients of a Mediterranean dietary pattern. 

## Figures and Tables

**Table 1:** Characteristics of Mediterranean diet pattern adherence indexes.

Food Groups	Mediterranean Diet Score (MDS) Trichopoulou, 2003	Alternative Mediterranean Diet (aMED) Fung, 2005	Relative Mediterranean Diet (rMED) Buckland, 2010	Dietary Score (DS) Panagiotakos, 2007	Literature-Based Adherence Score (LBAS) Sofi, 2014
Scoring criteria	Grams(g)/day	Rations/day	Energy density = g*1000 kcal/day	Rations/month	Grams(g)/day
Vegetables	0 points < median; 1 point ≥ median	0 points median; 1 point > median	Tertile 1 = 0 points; Tertile 2 = 1 point; Tertile 3 = 2 points	0 points=0, 1 point= 1–4, 2 points= 5–8, 3 points = 9–12, 4 points =13–18, 5 points =>18	0 points <100; 1 point 100–250; 2 points ≥250
Legumes	0 points < median; 1 point ≥ median	0 points ≤ median; 1 point > median	Tertile 1 = 0 points; Tertile 2 = 1 point; Tertile 3 = 2 points	0 points=0, 1 point= 1–4, 2 points= 5–8, 3 points = 9–12, 4 points =13–18, 5 points =>18	0 points <70; 1 point 70–140; 2 points ≥140
Fruit	(Included nuts) 0 points < median; 1 point ≥ median	0 points ≤ median; 1 point > median	Tertile 1 = 0 points; Tertile 2 = 1 point; Tertile 3 = 2 points	0 points=0, 1 point= 1–4, 2 points= 5–8, 3 points = 9–12, 4 points =13–18, 5 points =>18	0 points <150; 1 point 150–300; 2 points ≥300
Nuts	Included in fruit group	0 points ≤ median; 1 point > median	Included in fruit group	Not included	Not included
Fish	0 points < median; 1 point ≥ median	0 points ≤ median; 1 point > median	Tertile 1 = 0 points; Tertile 2 = 1 point; Tertile 3 = 2 points	0 points=0, 1 point= 1–4, 2 points= 5–8, 3 points = 9–12, 4 points =13–18, 5 points =>18	0 points <100; 1 point 100–250; 2 points ≥250
Cereals	0 points < median; 1 point ≥ median	(only whole grain) 0 points ≤ median; 1 point > median	Tertile 1 = 0 points; Tertile 2 = 1 point; Tertile 3 = 2 points	0 points=0, 1 point= 1–4, 2 points= 5–8, 3 points = 9–12, 4 points = 13–18, 5 points =>18	0 points <130; 1 point 130–195; 2 points ≥195
Scoring criteria	Grams(g)/day	Rations/day	Energy density = g*1,000 kcal/day	Rations/month	Grams(g)/day
Meat	(Poultry included) 1 point < median; 0 point ≥ median	(Red and processed meat) 0 points ≥ median; 1 point < median	(All kinds of meat) Tertile 1 = 2 points; Tertile 2 = 1 point; Tertile 3 = 0 points	(Red meat) 5 points = 0, 4 points = 1–4, 3 points = 5–8, 2 points = 9–12, 1 point = 13–18, 0 points ≥ 18	(Red and processed meat) 2 points <80; 1 point 80–120; 0 points ≥120
Dairy products	1 point < median; 0 point ≥ median	Not included	Tertile 1 = 2 points; Tertile 2 = 1 point; Tertile 3 = 0 points	(Dairy products with fats) 5 points = 0, 4 points = 1–4, 3 points = 5–8, 2 points = 9–12, 1 point = 13–18, 0 points ≥ 18	2 points <180; 1 point 180–270; 0 points ≥270
Mono/saturated fats ratio	0 points < median; 1 point ≥ median	0 points ≤ median; 1 point > median	Not included	Not included	Not included
Alcohol	Woman: 1 point → 5–25 g/day Man: 1 point → 10–50 g/day	Woman: 1 point → 5–15 g/day Man: 1 point → 10–25 g/day	Woman = 5–25 g/day and Man = 10–50 g/day (2 points) and <or> this quantity = 0 points	5 points ≤ 300, 4 points = 300, 3 points = 400, 2 points = 500, 1 point = 600, 0 points = 700 or 0	1 point <12; 2 point 12–24; 0 points ≥24
Potatoes	Included in vegetables group	Not included	Not included	0 points = 0, 1 point = 1–4, 2 points = 5–8, 3 points = 9–12, 4 points = 13–18, 5 points ≥ 18 (rations/week)	Not included
Olive oil cooking	Included in mono/saturated fats ratio group	Included in mono/saturated fats ratio group	Included in mono/saturated fats ratio group	0 points= never, 1 point = hardly ever; 2 points ≤ 1, 3 points = 1–3, 4 points = 3–5, 5 points= daily	0 points <0.1; 1 point 0.1–0.99; 2 points ≥1
Poultry	Included in meat group	Not included	Included in meat group	5 points =0, 4 points = 1–4, 3 points = 5–8, 2 points = 9–12, 1 point = 13–18, 0 points ≥ 18	Not included
Score ranged	0–9 points	0–9 points	0–18 points	0–55 points	0–18 points
Adherence categories	Low = 0–3 points Medium = 4–5 points High ≥ 6 points	Low = 0–3 points Medium = 4–5 points High ≥ 6 points	Low = 0–6 points Medium = 7–10 points High = 11–18 points	Low = tertile 1 Medium = tertile 2 High = tertile 3	Low ≤9 points Medium 9–11 points High ≥11 points

**Table 2:** Descriptive analysis of Mediterranean diet pattern adherence indexes (*n* = 3640).

		MDS	aMED	rMED	DS	LBAS
Sex						
Men	Mean (SD)	4.3 (1.7)	4.0 (1.8)	8.9 (3.3)	34.3 (4.4)	9.3 (2.3)
	Min–Max	(0–9)	(0–9)	(0–17)	(11–48)	(2–16)
	P25,50,75	(3–4–5)	(3–4–5)	(7–9–11)	(32–34–37)	(8–9–11)
Women	Mean (SD)	4.3 (1.6)	4.1 (1.8)	8.7 (3.1)	35.0 (4.2)	9.6 (2.0)
	Min–Max	(0–9)	(0–9)	(0–18)	(16–49)	(3–15)
	P25,50,75	(3–4–5)	(3–4–5)	(7–9–11)	(32–35–38)	(8–10–11)
Education level						
Less than primary	Mean (SD)	4.2 (1.7)	3.8 (1.7)	8.5 (3.3)	34.2 (4.8)	9.7 (2.3)
	Min–Max	(0–9)	(0–8)	(0–17)	(11–48)	(2–15)
	P25,50,75	(3–4–5)	(3–4–5)	(6–9–11)	(32–34–37)	(8–10–11)
Primary school	Mean (SD)	4.4 (1.6)	4.2 (1.7)	9.1 (3.2)	34.7 (4.2)	9.6 (2.2)
	Min–Max	(0–9)	(0–9)	(0–17)	(17–48)	(2–16)
	P25,50,75	(3–5–6)	(3–4–5)	(7–9–11)	(32–35–37)	(8–10–11)
Secondary	Mean (SD)	4.1 (1.7)	4.0 (1.8)	8.6 (3.2)	34.4 (4.3)	9.3 (2.1)
	Min–Max	(0–9)	(0–9)	(0–17)	(16–49)	(2–16)
	P25,50,75	(3–5–6)	(3–4–5)	(6–9–11)	(32–35–37)	(8–9–11)
University	Mean (SD)	4.2 (1.7)	4.1 (1.7)	8.9 (3.2)	35.0 (4.2)	9.4 (2.0)
	Min–Max	(0–9)	(0–9)	(0–18)	(16–49)	(3–15)
	P25,50,75	(3–4–5)	(3–4–5)	(7–9–11)	(32–35–38)	(8–9–11)
Total						
	Mean (SD)	4.3 (1.7)	4.1 (1.8)	8.8 (3.2)	34.6 (4.3)	9.4 (2.1)
	Min–Max	(0–9)	(0–9)	(0–18)	(11–49)	(2–16)
	P25,50,75	(3–4–5)	(3–4–5)	(7–9–11)	(32–34–37)	(8–9–11)

MDS: Mediterranean Diet Score [11]; aMED: alternative Mediterranean Diet [24]; rMED: relative Mediterranean Diet [25]; DS: Dietary Score [26]; LBAS: Literature-based adherence Score [20]. Min: minimum; Max: maximum; P25, 50, 75: 25th, 50th, and 75th percentiles.

**Table 3:** Degree of agreement among the adherence to a Mediterranean diet indexes according to the level of adherence: low, medium and high.

		Low Adherence				Medium Adherence				High Adherence			
Indexes		aMED	DS	rMED	LBAS	aMED	DS	rMED	LBAS	aMED	DS	rMED	LBAS
		*n* (%)	*n* (%)	*n* (%)	*n* (%)	*n* (%)	*n* (%)	*n* (%)	*n* (%)	*n* (%)	*n* (%)	*n* (%)	*n* (%)
	**Low**	935 (78.2)	580 (48.5)	569 (47.6)	751 (62.8)	250 (20.9)	368 (30.8)	535 (44.7)	367 (30.7)	11 (0.92)	248 (20.7)	92 (7.7)	78 (6.5)
**MDS**	**Medium**	443 (28.1)	417 (26.4)	287 (18.2)	374 (23.7)	896 (56.8)	555 (35.2)	781 (49.5)	727 (46.1)	239 (15.1)	606 (38.4)	510 (32.3)	477 (30.2)
	**High**	20 (2.3)	97 (11.2)	19 (2.2)	43 (5.0)	294 (33.9)	267 (30.8)	307 (35.4)	219 (25.3)	552 (63.7)	502 (58.0)	540 (62.4)	604 (69.7)
	**Low**	837 (71.7)	588 (50.3)	523 (44.8)		289 (24.7)	326 (27.9)	496 (42.5)		42 (3.6)	254 (21.7)	149 (12.8)	
**LBAS**	**Medium**	440 (33.5)	346 (26.3)	267 (20.3)		650 (49.5)	483 (36.8)	638 (48.6)		223 (16.9)	484 (36.9)	408 (31.1)	
	**High**	121 (10.4)	160 (13.8)	85 (7.3)		501 (43.2)	381 (32.9)	489 (42.2)		537 (46.3)	618 (53.3)	585 (50.5)	
	**Low**	629 (71.9)	442 (50.5)			220 (25.1)	239 (27.3)			26 (3.0)	194 (22.2)		
**rMED**	**Medium**	635 (39.1)	461 (28.4)			717 (44.2)	544 (33.5)			271 (16.7)	618 (38.1)		
	**High**	134 (11.7)	191 (16.7)			503 (44.1)	407 (35.6)			505 (44.2)	544 (47.6)		
	**Low**	706 (64.5)				327 (29.9)				61 (5.6)			
**DS**	**Medium**	430 (36.1)				523 (43.9)				237 (19.9)			
	**High**	262 (19.3)				590 (43.5)				504 (37.2)			

MDS: Mediterranean diet score [11]; aMED: alternative Mediterranean diet [24]; rMED: relative Mediterranean diet [25]; DS: dietary score [26]; LBAS: Literature-based adherence Score [20].

**Table 4:** Reliability among the indexes of adherence to the Mediterranean diet pattern (three categories: low, medium and high adherence).

	Indexes	aMED	rMED	DS	LBAS
	Correlation coefficient (95% CI)	0.75 (0.74–0.77)	0.56 (0.55–0.59)	0.42 (0.40–0.45)	0.65 (0.63–0.67)
MDS	KAPPAa	0.56 (0.55–0.59)	0.37 (0.36–0.39)	0.25 (0.24–0.27)	0.45 (0.44–0.47)
	KAPPA^2b	0.67 (0.66–0.68)	0.49 (0.48–0.51)	0.33 (0.31–0.35)	0.56 (0.55–0.58)
	Correlation coefficient (95% CI)	1	0.56 (0.54–0.59)	0.52 (0.49–0.54)	0.62 (0.60–0.64)
aMED	KAPPAa		0.36 (0.35–0.37)	0.30 (0.28–0.32)	0.43 (0.42–0.45)
	KAPPA^2b		0.47 (0.45–0.49)	0.39 (0.37–0.40)	0.53 (0.52–0.56)
	Correlation coefficient (95% CI)		1	0.35 (0.32–0.38)	0.49 (0.46–0.51)
rMED	KAPPAa			0.19 (0.18–0.21)	0.30 (0.29–0.32)
	KAPPA^2b			0.26 (0.25–0.28)	0.40 (0.38–0.42)
	Correlation coefficient (95% CI)			1	0.45 (0.42–0.47)
DS	KAPPAa				0.26 (0.25–0.28)
	KAPPA^2b				0.33 (0.30–0.36)

MDS: Mediterranean diet score [11]; aMED: alternative Mediterranean diet [24]; rMED: relative Mediterranean diet [25]; DS: dietary score [26]; LBAS: Literature-based adherence Score [20]. A correlation coefficient *R* higher than 0.70 was considered as a strong correlation; from 0.5 to 0.7 as a moderate correlation; and <0.5 as a weak correlation. 95% CI: 95% Confidence Interval. ^a^ Lineal weighting Kappa. Weighting matrix (1–0.5–0). ^b^ Quadratic weighting Kappa. Weighting matrix (1–0.75–0).

## Supplementary Materials

The following are available online at https://www.mdpi.com/2072-6643/11/3/488/s1, Table S1: Socio-demographic characteristics of the total of controls and the FFQ-controls included in our study; Table S2: Percentage and confidence intervals of the levels of adherence to the Mediterranean diet.
